# Overexpression of AS*vicR* combined with the antibacterial monomer DMAHDM interferes with the VicRK two-component system to attenuate the cariogenicity of *Streptococcus mutans*

**DOI:** 10.3389/fcimb.2026.1793140

**Published:** 2026-03-05

**Authors:** Yuting Sun, Han Du, Xuele Pan, Zheng Wang, Xinyi Zhang, Ruiqi Yang, Hong Chen, Deqin Yang

**Affiliations:** 1 Department of Endodontics, The Afffliated Stomatological Hospital of Chongqing Medical University, Chongqing, China; 2 Stomatological Hospital of Chongqing Medical University, Chongqing Key Laboratory of Oral Diseases and Biomedical Sciences, Chongqing, China; 3 Chongqing Municipal Key Laboratory of Oral Biomedical Engineering of Higher Education, Chongqing, China; 4 Chongqing Municipal Health Commission Key Laboratory of Oral Biomedical Engineering, Chongqing, China; 5 Department of Endodontics, Shanghai Stomatological Hospital & School of Stomatology, Fudan University, Shanghai, China; 6 Shanghai Key Laboratory of Craniomaxillofacial Development and Diseases, Fudan University, Shanghai, China

**Keywords:** antisense vicR (ASvicR), cariogenicity, dimethylaminohexadecylmethacrylate (DMAHDM), *Streptococcus mutans*, VicRK TCS

## Abstract

**Background:**

*Streptococcus mutans* (*S. mutans*) is a primary cariogenic pathogen responsible for acid production, exopolysaccharides (EPS) production and biofilm formation. Two-component systems (TCS) regulate EPS metabolism, especially the VicRK TCS. Overexpression of antisense *vicR* (AS*vicR*) can reduce EPS production and thereby weaken the cariogenicity of *S. mutans.* Although the antimicrobial monomer dimethylaminohexadecyl methacrylate (DMAHDM) exhibits potent antibacterial properties, mature *S. mutans* biofilms can protect themselves by extracellular matrix. Emerging evidence suggests that genetic intervention enhances drug efficacy, yet the underlying regulatory mechanisms remain largely unexplored.

**Objective:**

To investigate the chemical–genetic cooperative antibiofilm strategy inhibition and mechanisms of AS*vicR* overexpression combined with DMAHDM on *S. mutans* biofilm formation, acid and EPS metabolism, and cariogenicity through the VicRK system.

**Methods:**

The minimal inhibitory concentration and minimal bactericidal concentration of DMAHDM and chlorhexidine were determined. Biofilm properties were evaluated via biomass assessment, EPS quantification, lactate production measurement, and colony-forming unit counting. Biofilm structures were examined by scanning electron microscopy. Mechanisms were investigated using RT-qPCR, zymography, and western blot. Rat caries model was employed to assess caries formation under different treatment conditions.

**Results:**

The AS*vicR* strain exhibited an approximate 2-fold increase in susceptibility to DMAHDM and chlorhexidine. The combination treatment reduced biofilm CFU by approximately 4 log units, significantly lowered lactate and EPS levels, and resulted in a loose, porous biofilm structure. The expression levels of cariogenic virulence factors as well as the VicRK TCS genes and proteins were significantly downregulated. *In vivo*, the combined treatment reduced the overall caries severity score to 12.7% of the control group (p<0.05) without observing any systemic adverse effects.

**Conclusion:**

The strategy of combining AS*vicR* overexpression with DMAHDM effectively modulates EPS metabolism and cariogenicity in *S. mutans* by interfering with the VicRK TCS, providing a potential therapeutic approach for clinical caries management.

## 1 Introduction

Dental caries, a chronic and progressive infectious disease of the hard tissues of teeth, intrinsically links to the accumulation of dental plaque biofilms (Selwitz et al., 2007). Beyond its localized destruction, emerging evidence indicates that untreated caries can precipitate severe oral sequelae and systemic pathologies, making effective early prevention and intervention a critical public health priority (Liu et al., 2023). *Streptococcus mutans* (*S. mutans*) is widely held as a primary pathogen causing caries, largely relying on its ability to converting dietary sucrose into exopolysaccharides (EPS) (Collaborators, 2025; Jiang et al., 2025). This EPS matrix enhances biofilm density and adhesion, constructing a physical fortress that shields bacteria from antibiotic (Hemadi et al., 2017). Consequently, as the structural core and energy reserve of the biofilm, EPS is pivotal in conferring resistance to therapeutic interventions (Emmanuelli et al., 2023; Du et al., 2025).

The virulence and adaptive capabilities of *S. mutans* are intricately regulated by two-component signal transduction systems (TCSs). Among the roughly 14 TCSs identified in streptococci, the VicRK system is indispensable for cell wall homeostasis, stress tolerance, and, crucially, EPS metabolism (Kawada-Matsuo and Komatsuzawa, 2017; Lei et al., 2020). Upon environmental stimulation, the histidine kinase VicK phosphorylates the response regulator VicR (Sun et al., 2023), which directly modulates the expression of the key polysaccharide synthesis genes, including *gtfB*/*C*/*D* and *dexA*. However, the investigation of VicR has been historically challenged by its status as an essential gene, precluding the generation of viable knockout strains. To circumvent this, recent studies have validated a knockdown strategy using a specific non-coding antisense RNA, AS*vicR* (Tian et al., 2022). By forming a double-stranded RNA complex with the target *vicR* mRNA, AS*vicR* triggers degradation via RNase III, interfering with VicR translation and subsequently suppressing downstream EPS synthesis and biofilm maturation (Lei et al., 2023; Deng et al., 2021).

Clinically, caries management has shifted towards a prevention-oriented non-surgical interventions in accordance with the American Dental Association (ADA) guidelines. Current strategies often combine biological agents to restore microbial homeostasis with chemical agents to promote remineralization (Chen et al., 2025).While effective for early-stage lesion reversal, these approaches often lack the potency to disrupt established, mature biofilms or sustain the inhibition of virulent *S. mutans* populations. This limitation underscores the urgent need for strategies that integrate precise “microbial regulatory targets” with advanced “antimicrobial materials.”

Among existing antibacterial materials, chlorhexidine (CHX) remains the most widely used antimicrobial agent in clinical dentistry owing to its well-documented efficacy against *S. mutans.* CHX is commonly adopted as a benchmark in *in vitro* evaluations of antibiofilm and anti-cariogenic strategies. However, its long-term efficacy is limited. Quaternary ammonium methacrylates (QAMs) have emerged as promising candidates (Chen et al., 2024; Wang et al., 2022). Cationic monomers, particularly dimethylaminohexadecyl methacrylate (DMAHDM) with its 16-carbon alkyl chain, can be copolymerized into resin matrices to provide durable, non-leaching, contact-dependent antibacterial activity (Su et al., 2020). DMAHDM achieves contact-based killing of bacteria, primarily by disrupting the bacterial cell membranes. However, *S. mutans* can form dense biofilm encapsulated by extracellular matrix, which serves as a barrier for the diffusion of antibacterial drugs and thereby enhances the drug resistance of the biofilm (Fanfoni et al., 2021; Balhaddad et al., 2021). We previously demonstrated that combining DMAHDM with a strain overexpressing the negative regulator *gcrR* yielded a significant effect. This finding validates the concept that targeting essential regulatory genes can sensitize bacteria to antimicrobial materials (Chen et al., 2024).

Building on our prior work characterizing the VicRK system—antisense *vicK* (AS*vicK*) overexpression reduced EPS and sensitized biofilms to DMAHDM—we propose that targeting the essential regulator VicR offers a superior avenue for disruption. VicR directly controls the transcriptional network of glucosyltransferases and dextranases, making it a potent target for interfering with biofilm resistance (Yu et al., 2024). Here, we proposed a new combinatorial strategy: the integration of AS*vicR* overexpression with DMAHDM. We aim to systematically evaluate impact of this bio-material approach on *S. mutans* biofilm formation, EPS metabolism, and cariogenicity, thereby elucidating the underlying regulatory mechanisms and establishing a theoretical foundation for next-generation caries prevention therapies.

## 2 Materials and methods

### 2.1 Synthesis of DMAHDM

DMAHDM with >99% purity was acquired by reacting 2 - (dimethylamino) ethyl methacrylate with 1-bromododecane via a Menschutkin reaction (Chen et al., 2020).

### 2.2 Construction of the AS*vicR* strain

**Figure 1:**
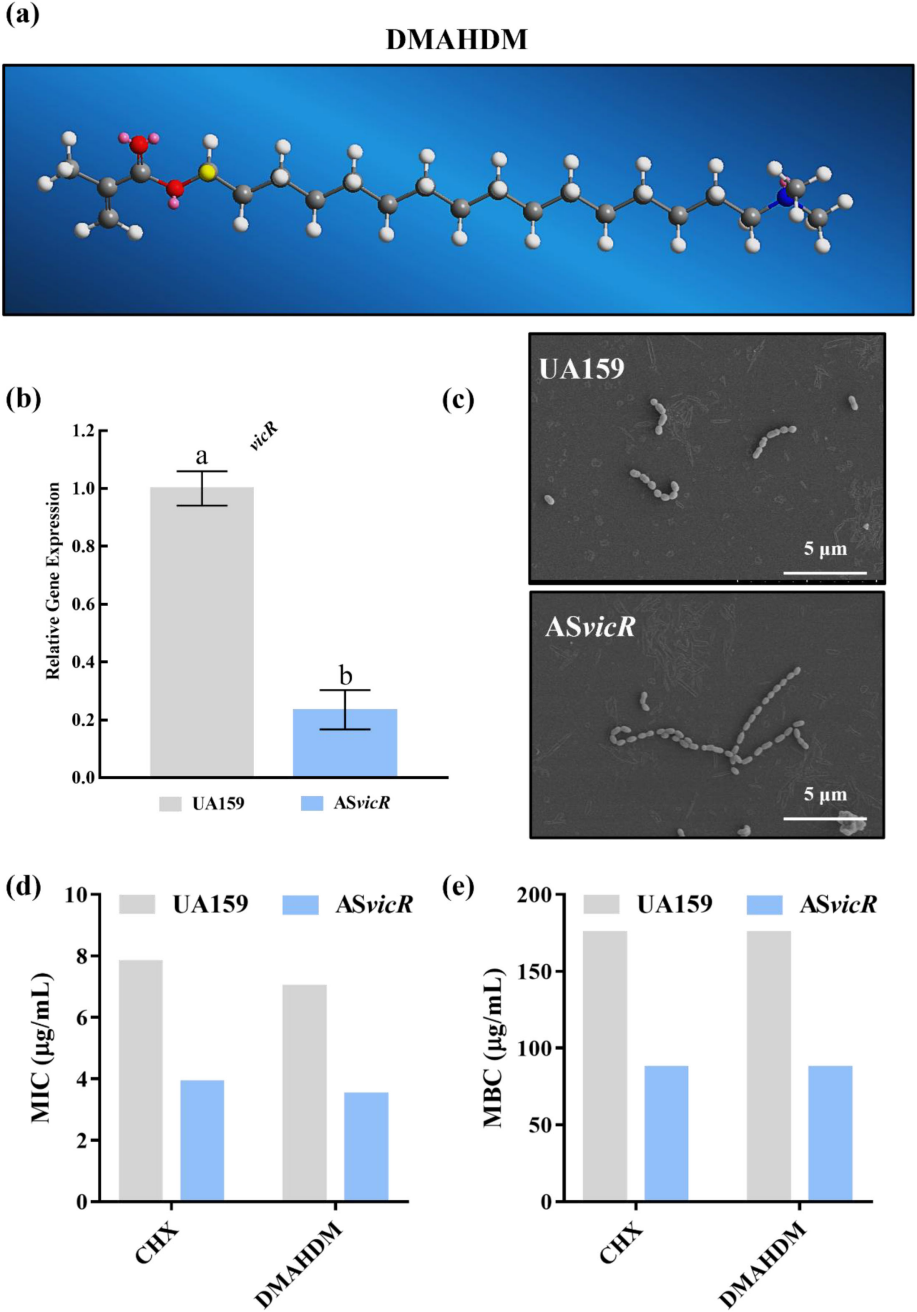
Validation of the AS*vicR* overexpression strain and its drug susceptibility compared to UA159. **(a)** Three-dimensional chemical structure of DMAHDM; **(b)** verification of *vicR* gene expression by RT-qPCR (n = 3); **(c)**morphology of planktonic bacteria observed by SEM; **(d)** MIC and **(e)** MBC-B of CHX and DMAHDM against the respective strains (n = 6). Dissimilar letters represent significant differences (p < 0.05).

The Ethics Committee of the School of Stomatology, Chongqing Medical University approved this research. The AS*vicR* gene was added to the pDL278 vector, and then the vector was transformed into the UA159 strain (ATCC: 700610). Verification was carried out using selective media and RT-qPCR (**Figures 1b, c**) (Lei et al., 2020). The strains and plasmids involved in our research are statement in **Supplementary Table 1**.

### 2.3 Determination of the values of minimum inhibitory concentration and minimum bactericidal concentration

CHX was included as a positive control in this study, and we established six experimental groups: UA159 + PBS, UA159 + CHX, UA159 + DMAHDM, AS*vicR* + PBS, AS*vicR* + CHX, and AS*vicR* + DMAHDM. For MIC, adjust the initial concentrations of DMAHDM and CHX were 112 μg/mL and 250 μg/mL, respectively (Gonzalez et al., 2013).For MBC, 24 hours biofilms medium was replaced with BHIS (BHI medium supplemented with 1% sucrose)medium containing serially diluted concentrations of DMAHDM or CHX, starting at 300 μg/mL (Wiegand et al., 2008). After 24 hours of cultivation, the biofilms were serially diluted and plated on agar to count the colony-forming unit (CFU) (Wang et al., 2017).

### 2.4 Culture conditions of sample

Parental strain (UA159) was inoculated in BHI, while the AS*vicR* strain was cultured in BHI supplemented with 1 mg/mL spectinomycin. Both strains were incubated under a mixed gas atmosphere (37 °C, 80% N_2_, 10% CO_2_, 10% H_2_) for 14–16 hours. The cultures were then grown to mid-logarithmic phase, after which the OD values were adjusted to be consistent (Sugai et al., 2023). The cultures were diluted in BHIS (10^7^; CFU/mL) and incubated for 24 hours to foster biofilm formation. Subsequently, BHIS was removed, and BHIS containing 87.5 μg/mL CHX or DMAHDM was added followed by 24-hour incubation.

### 2.5 Quantification of biofilm biomass by crystal violet assay

Crystal violet staining is performed after fixing with methanol. Acetic acid solution was used for elution to obtain the dye and a microplate reader was used measuring the OD values at 550 nm of the solution to quantify the biofilm biomass.

### 2.6 Determination of reactive oxygen species in biofilms

After washed with PBS, 48-hour mature biofilms were carefully scraped from the substrate and subjected to mild ultrasonication to achieve a homogeneous suspension. The diluted 2’,7’-dichlorodihydrofluorescein diacetate probe solution (Beyotime Biotechnology, China) was added, then the samples were incubated at 37 °C in the dark for 20–30 minutes. Blank and positive control groups were included in the experimental setup. A microplate reader was used to measured Fluorescence intensity.

### 2.7 Measurement of lactate production in biofilms

After rinsed by PBS, the biofilms from each group were added with buffered peptone water containing 0.2% sucrose. These samples were incubated for 3 hours to allow for acid production. A commercial Lactate Assay Kit (Jiancheng Bioengineering Institute, Nanjing, China) was applied to determine the lactate concentration in the supernatant.

### 2.8 Measurement of lactate dehydrogenase activity in biofilms

Biofilms in each group were gently washed two to three times with PBS. A commercial Lactate Dehydrogenase Activity Kit (Jiancheng Bioengineering Institute, Nanjing, China) was applied to determine the LDH activity.

### 2.9 Analysis of live/dead bacterial in biofilms by confocal laser scanning microscopy

48 hours Biofilms were stained with 2.5 μM SYTO 9 (Live bacteria, green)and 2.5 μM propidium iodide (dead bacteria, red) for 15 minutes in the absence of light (Liu et al., 2016). The stained biofilms were then examined using CLSM. For each sample, images were acquired from three randomly selected areas. The excitation wavelengths for SYTO 9 and PI were 485 nm and 617 nm, and the corresponding emission wavelengths were 498 nm and 617 nm, respectively. Z-stack images were obtained with a 1-μm axial step size. Three-dimensional biofilm reconstructions were generated using Leica Application Suite X (LAS X) software (Chen et al., 2022).

### 2.10 Examination of biofilm architecture by scanning electron microscopy

The biofilms grown on sterile round coverslips were fixed with 2.5% glutaraldehyde at 4 °C for 4 hours and then dehydrated through a graded ethanol series (Quintero-Yanes et al., 2025). Critical point drying with CO_2_ was performed, followed by gold sputter coating. The morphological characteristics of biofilms were examined by SEM (Inspect F50, FEI, USA), and representative images were captured. All experiments were conducted independently in triplicate.

### 2.11 Extraction and quantification of EPS

Water-soluble glucans (WSG)​ and water-insoluble glucans (WIG) were extraction (Lin et al., 2022) and tested through the anthrone-sulfuric acid method. Ang the concentration of polysaccharide was calculated based on the absorbance values of the samples (Lei et al., 2023; Lin et al., 2022).

### 2.12 Analysis of biofilm architecture by CLSM

1 μM Alexa Fluor^®^ 647 conjugate was added at the initial stage of biofilm formation to label EPS (Zhang et al., 2022). Biofilms were then stained with 50 μL of SYTO 9 nucleic acid stain for 15 minutes. The procedures for CLSM imaging, 3D reconstruction and analysis were identical to those used for the live/dead staining assay.

### 2.13 Analysis of gene expression by reverse transcription - quantitative polymerase chain reaction

Trizol method was used to extract total RNA of the 48-hour biofilms (Bustin, 2024). The PrimeScript™ RT reagent kit (TaKaRa, Japan) was applied to remove genomic DNA (gDNA) and synthesize complementary DNA (cDNA). RT-qPCR was then performed using TB Green™ Premix Ex Taq™ (TaKaRa, Japan). The expression of genes was calculated by comparative threshold cycle method. All experiments were conducted independently in triplicate. The primer sequences involved in this study are showed in **Supplementary Table 2**.

### 2.14 Zymographic analysis of glucosyltransferase activity in biofilms

Biofilms were collected and centrifuged for 10 minutes (13,000 rpm). Filtered the supernatant with 0.22-μm membrane and incubated it with absolute ethanol at −80 °C. The pellets were collected after centrifuged (25,000 rpm), and the protein concentrations were determined and adjusted to equal levels. After the addition of loading buffer, samples were subjected to electrophoresis and stained with Quick Coomassie Blue Staining Solution (Beyotime Biotechnology, China) for 1 hour, followed by destaining for 24 hours to visualize total protein (Lei et al., 2018; Liu et al., 2022).

### 2.15 Analysis of protein expression in biofilms by western blot

Protein samples were extracted from 48-hour biofilms with RIPA lysis buffer and homogenized. The proteins were collected after centrifugation, and determined concentration using the BCA method. 20 μg of protein was separated by SDS-PAGE and then transferred onto PVDF membranes. After blocked, the membranes were incubated overnight at 4 °C with primary antibodies (anti-VicR, anti-VicK, anti-Rnc, anti-DexA). Following being washed with TBST solution, the samples were incubated for 2 hours at room temperature with secondary antibody (goat anti-rabbit). The enhanced chemiluminescence substrate was used to observe the protein bands. Total protein loading was verified by Coomassie Brilliant Blue staining (Lei et al., 2018; Liu et al., 2022).

### 2.16 Establishment of a rat caries model

An animal model was established following the previous protocol in accordance with the ARRIVE guidelines. The experimental timeline is illustrated in **Supplementary Figure 3**. Eighty 21-day-old specific-pathogen-free (SPF) Sprague-Dawley (SD) rats were randomly divided into eight groups, with 10 rats in each group: UA159 + PBS, UA159 + CHX, UA159 + DMAHDM, AS*vicR* + PBS, AS*vicR* + CHX, AS*vicR* + DMAHDM, Blank (sterile PBS), and Negative Control (BHI medium). All rats were housed in the animal facility of the Affiliated Stomatological Hospital of Chongqing Medical University. During the entire experiment period, these rats were maintained on a cariogenic diet (Keyes 2000# diet; Dasuo, Chengdu, China) and sterile water supplemented with 5% sucrose. The oral streptococcal flora status of the rats was assessed using MSA media. 0.1% ampicillin, 0.1% chloramphenicol, and 0.1% carbenicillin were used to suppress the native oral flora for three days, followed by a one-day antibiotic-free period. Oral streptococcal status was re-assessed thereafter. Bacterial cultures of each strain, harvested at the mid-logarithmic growth phase, were applied to the mandibular molar surfaces of the rats and 200 μL of bacterial suspension was inoculated. Food and water were withheld for 3 hours post-inoculation. This inoculation procedure was performed once daily for seven consecutive days. To verify successful colonization by *S. mutans*, dental plaque was collected from the rats. Genomic DNA was extracted and subjected to PCR amplification. Then, bacterial colonization was determined through agarose gel electrophoresis. Following colonization, the tooth surfaces were treated once daily with the respective assigned agents (PBS, CHX, or DMAHDM) applied with a brush for 5 minutes, over a period of 5 days. These rats were then maintained for three weeks and subject to daily health check and weighed weekly. The rats were euthanized by CO_2_ asphyxiation after the experiment. The mandibles and organs (heart, liver, spleen, lungs, kidneys) were harvested. After fixed in 4% paraformaldehyde, the organ samples were processed for hematoxylin and eosin (HE) staining, and the histological sections were examined under a microscope. For caries evaluation, after fixed in 4% paraformaldehyde, the mandibles were stained with 0.4% ammonium purpurate for 6 hours. Caries lesions on the smooth surfaces were examined and scored under a stereomicroscope. Subsequently, each molar was sectioned mesiodistally, and caries lesions on the occlusal surfaces were evaluated. The severity of the carious lesions was graded according to the Keyes criteria: E (Enamel limitation, smooth surface caries), Ds (Dentin slight, occlusal caries limited to enamel), Dm (Dentin medium, occlusal caries extending into the outer half of the dentin, exceeding one-quarter but less than three-quarters of the dentin thickness), and Dx (Dentin extensive, occlusal caries involving the inner half of the dentin, exceeding three-quarters of the dentin thickness). Scoring was performed independently by two examiners who were both unaware of group assignments. Two specimens from each group were observed using a SEM (Inspect F50, FEI, USA). The sample processing procedure was the same as that for the previous biofilm samples. The morphological characteristics of the bacterial biofilms on the molar surfaces were examined using a SEM and representative images were captured.

### 2.17 Statistical analysis

Statistical analysis was performed with SPSS 22.0 (SPSS Inc., Chicago, IL, USA). Intergroup differences were statistically examined via one-way analysis of variance (ANOVA), based on the homogeneity of variances afterwards. If the ANOVA indicated a significant difference, *post-hoc* comparisons between groups were conducted. The method used was Tukey’s honestly significant difference test. It’s considered to have statistical significance when the p-value is less than 0.05.

## 3 Results

### 3.1 AS*vicR* alters the susceptibility of *S. mutans* to drugs

The chemical structure of DMAHDM is shown in **Figure 1a**. Successful construction of the AS*vicR* overexpression strain was verified by RT-qPCR, which confirmed the downregulated *vicR* gene expression in the mutant strain during the growth phase relative to the wild-type strain (**Figure 1b**; n = 3). Both planktonic strains were further verified by SEM, and they typically presented a spherical morphology with smooth surface and arranged in chains of variable length (**Figure 1c**). For the wild-type UA159 strain, MIC values for CHX and DMAHDM were 7.8125 μg/mL and 7 μg/mL, respectively. The MBC-B for both agents against UA159 was 175 μg/mL. In contrast, the AS*vicR* strain showed lower MIC values of 3.90625 μg/mL for CHX and 3.5 μg/mL for DMAHDM (**Figure 1d**). The MBC-B values for both compounds against the AS*vicR* strain were 87.5 μg/mL (**Figure 1e**). The MBC-B values for both compounds against the AS*vicR* strain were 87.5 μg/mL. These results indicate that the AS*vicR* strain exhibited significantly higher susceptibility, in both planktonic and biofilm states, to both DMAHDM and CHX than the parent strain.

### 3.2 AS*vicR* combined with DMAHDM inhibits growth and activity of *S. mutans*

**Figure 2:**
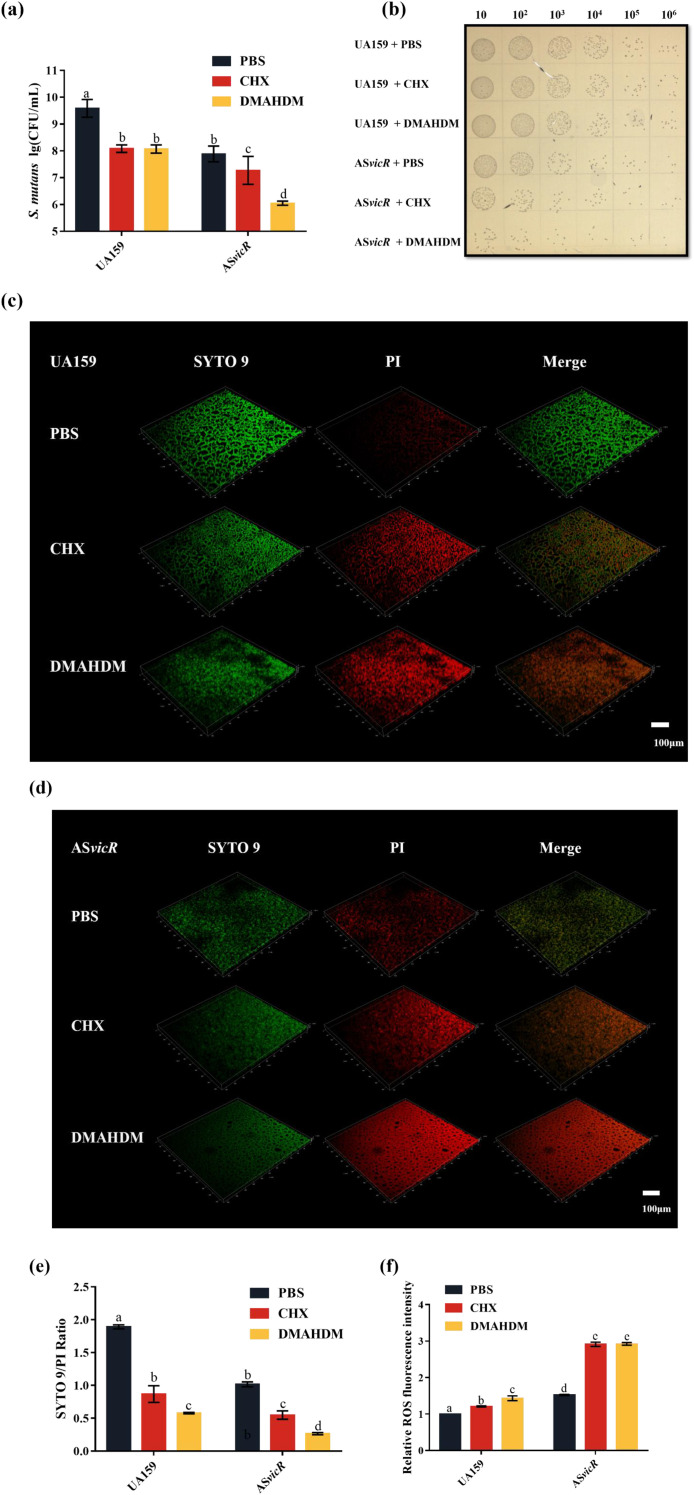
Combined effect of AS*vicR* overexpression and DMAHDM on the viability of *S. mutans*. **(a)** CFU of pre-formed 48-hour biofilms of UA159 and AS*vicR* strains treated with the indicated agents (n = 6) and **(b)** images; **(c)** fluorescence staining of biofilm live/dead bacteria in each group of UA159 strain and **(d)** AS*vicR* strains (green: live bacteria; red: dead bacteria); **(e)** SYTO 9/PI fluorescence ratio (n = 3). **(f)** intracellular ROS levels in planktonic cells of UA159 and AS*vicR* strains after treatment (n = 6). Dissimilar letters represent significant differences (p < 0.05).

The AS*vicR* group without drug treatment had reduced CFU counts compared to the UA159. Upon treatment with CHX or DMAHDM, the AS*vicR* strain revealed a more significant decrease in CFU than the wild-type strain, with the AS*vicR* + DMAHDM group exhibiting the lowest CFU counts (**Figures 2a, b**; n = 6). Most antibacterial drugs can induce excessive generation of intracellular ROS in bacteria, causing oxidative stress damage and thereby inhibiting bacterial proliferation or directly killing the bacteria. These changes in ROS levels can affect the quorum sensing system, the metabolism of EPS, and the expression of acid tolerance-related genes. We found that the ROS level was highest in the AS*vicR* + DMAHDM group (**Figure 2f**), indicating that DMAHDM induces oxidative stress, potentially disrupting cellular metabolism and enhancing its antibacterial effect. Representative 3D images of live/dead cells of 48-hour biofilms showed that, in the absence of drug treatment, biofilms from both groups were mostly composed of live bacteria (**Figures 2c, d**). The weaker green fluorescence in the AS*vicR* control group suggests a thinner biofilm with lower bacterial density (**Figure 2e**). After DMAHDM treatment, both strains showed a shift towards a predominantly dead bacterial population, more pronounced in the AS*vicR* group. The AS*vicR* + DMAHDM group exhibited the highest dead-to-live bacteria ratio, confirming the increased susceptibility of the AS*vicR* strain to DMAHDM.

### 3.3 Combined effect of AS*vicR* overexpression and DMAHDM on cariogenic virulence factors of biofilm

**Figure 3:**
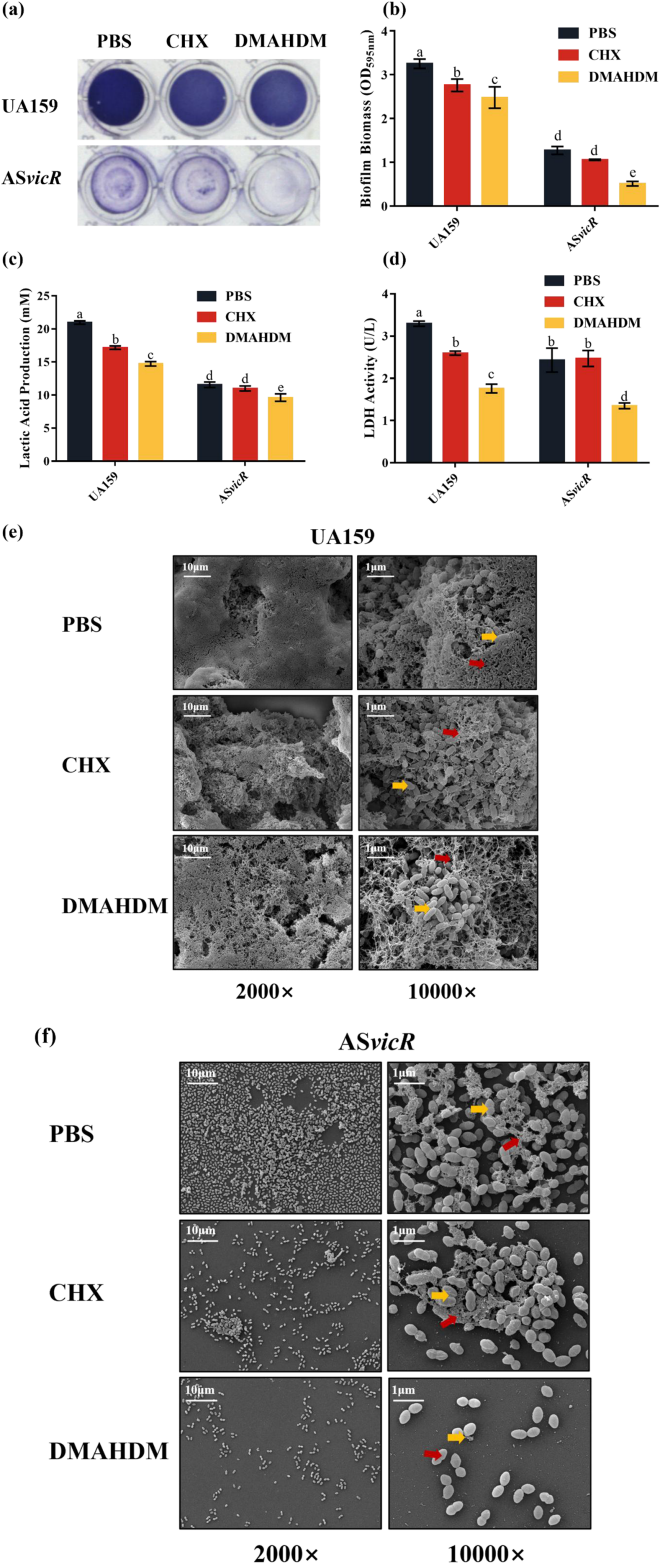
AS*vicR* overexpression enhances the antibacterial effects of DMAHDM on cariogenic virulence factors in biofilms. **(a)** Crystal violet staining of biofilms after various treatments; **(b)** quantitative analysis of biofilm biomass (n = 6); **(c)** lactate production and **(d)** LDH activity in treated biofilms (n = 6); SEM images showing the architectures of UA159 **(e)** and AS*vicR***(f)** biofilms, with bacterial cells and EPS indicated by yellow and red arrows, respectively. Dissimilar letters represent significant differences (p < 0.05).

AS*vicR* overexpression combined with DMAHDM affects virulence factors, including biofilm biomass and architecture and lactate content. Crystal violet staining showed a clear treatment effect on biofilms (**Figure 3a**). The AS*vicR* + PBS group exhibited significantly decreased biofilm biomass compared to the UA159 + PBS group. CHX treatment had no significant effect, whereas DMAHDM resulted in the most substantial reduction (**Figure 3b**). Lactate production was reduced by 54.9% in the AS*vicR* + DMAHDM group compared to the UA159 + PBS group (**Figure 3c**). A significant decrease in LDH activity confirmed impaired acidogenesis (**Figure 3d**). SEM images revealed structural differences between strains. The UA159 + PBS group formed dense biofilms with abundant extracellular matrix (**Figure 3e**). Both CHX and DMAHDM caused moderate reductions in bacterial clusters and matrix. In the AS*vicR* strain, PBS and CHX groups showed sparse biofilms with reduced matrix. Notably, DMAHDM prevented significant biofilm formation in the AS*vicR* strain, leaving dispersed bacterial cells with minimal cohesion (**Figure 3f**). These results demonstrate that combining AS*vicR* overexpression with DMAHDM effectively disrupts biofilm formation and matrix production in *S. mutans*.

### 3.4 AS*vicR* combined with DMAHDM inhibits EPS production and architecture of biofilm

**Figure 4:**
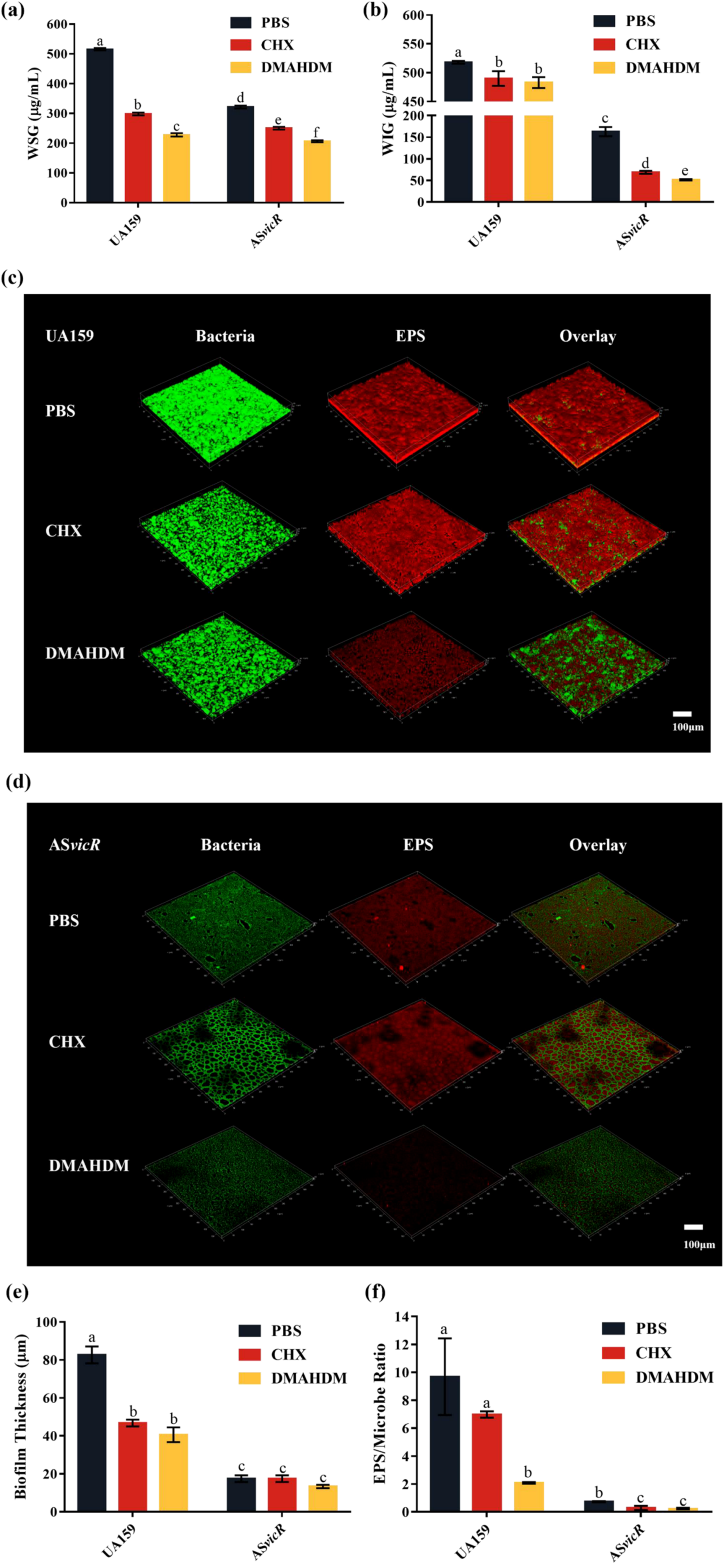
Combined treatment with AS*vicR* and DMAHDM suppresses EPS production in biofilms. **(a)** WSG and **(b)** WIG contents in biofilms (n = 6); CLSM images of **(c)** UA159 and **(d)** AS*vicR* strain biofilms stained with Alexa Fluor 647-conjugated dextran (red: EPS) and SYTO 9 (green: bacteria); **(e)** biofilm thickness of UA159 and AS*vicR* strains (n = 3); **(f)** EPS/microbe ratio (n = 3). Dissimilar letters represent significant differences (p < 0.05).

WSG promote bacterial adhesion and initial biofilm formation, while WIG form a three-dimensional cross-linked network. Given that both of them are key factors in dental caries development, we quantified their contents in the biofilms. Compared with UA159, the AS*vicR* biofilms contained less WSG and WIG, and this reduction was further enhanced after treatment with DMAHDM or CHX (**Figures 4a, b**). Notably, the AS*vicR* + DMAHDM group has lower content of WIG, which aligns with the SEM observations showing disrupted biofilm formation in this group. Furthermore, CLSM analysis of biofilm architecture and EPS content revealed that the combination treatment group exhibited a looser biofilm structure (**Figures 4c, d**), a reduced capacity for EPS production (**Figure 4e**) and a decreased biofilm thickness (**Figure 4f**) compared to the other groups.

### 3.5 The combination of AS*vicR* and DMAHDM regulates the expression of genes and proteins related to EPS metabolism

**Figure 5:**
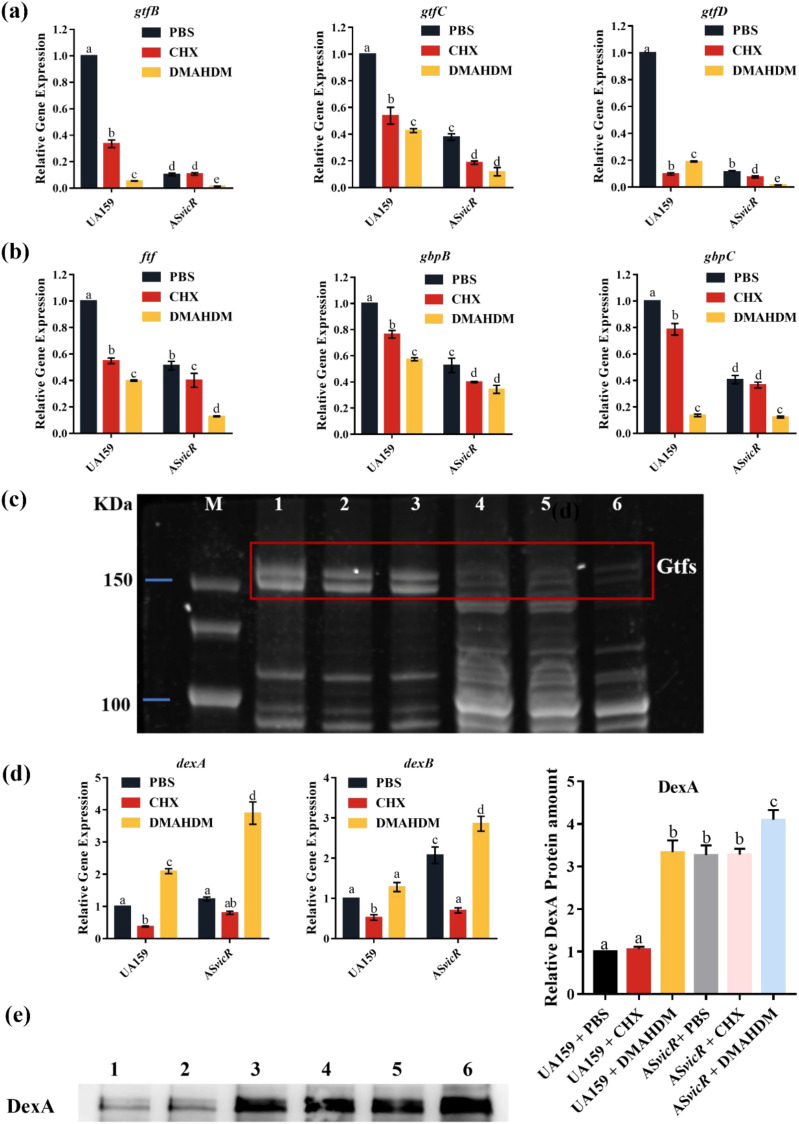
Analysis of gene and protein expression related to glucose metabolism. **(a)** Gene expression of *gtfB*/*C*/*D*, **(b)***ftf*, and *gbpB*/*C* analyzed by RT-qPCR (n = 3); **(c)** Gtfs (GtfB/C/D)enzyme activity in biofilms, M: marker, 1: UA159 + PBS, 2: UA159 + CHX, 3: UA159 + DMAHDM, 4: AS*vicR* + PBS, 5: AS*vicR* + CHX, 6: AS*vicR* + DMAHDM; **(d)** expression of *dexA/B* detected by RT- qPCR; **(e)** expression level of DexA detected by western blot (n = 3). Coomassie-stained gels confirmed equal protein loading among samples (see **Supplementary Figure 1**). Dissimilar letters represent significant differences (p < 0.05), 1: UA159 + PBS, 2: UA159 + CHX, 3: UA159 + DMAHDM, 4: AS*vicR* + PBS, 5: AS*vicR* + CHX, 6: AS*vicR* + DMAHDM.

RT-qPCR analysis revealed that compared to the UA159 + PBS group, the expression of glucosyltransferase genes *gtfB*/*C*/*D* responsible for synthesizing EPS was significantly reduced in AS*vicR* + DMAHDM group (**Figure 5a**). Concurrently, the fructosyltransferase gene *ftf* and the glucan-binding protein genes *gbpB* and *gbpC* were also markedly downregulated (**Figure 5b**). In contrast, the expression of endodextranase genes *dexA* and *dexB*, which hydrolyze EPS, was significantly increased (**Figure 5d**), along with a notable rise in DexA protein expression (**Figure 5e**). To link expression changes with functional outcomes in EPS synthesis, glucosyltransferase activity was evaluated. Further analysis of Gtfs enzyme activity showed a significant decline in the AS*vicR* + DMAHDM group (**Figure 5c**).

### 3.6 The combination of AS*vicR* and DMAHDM regulates the expression of genes and proteins of VicRK TCS

**Figure 6:**
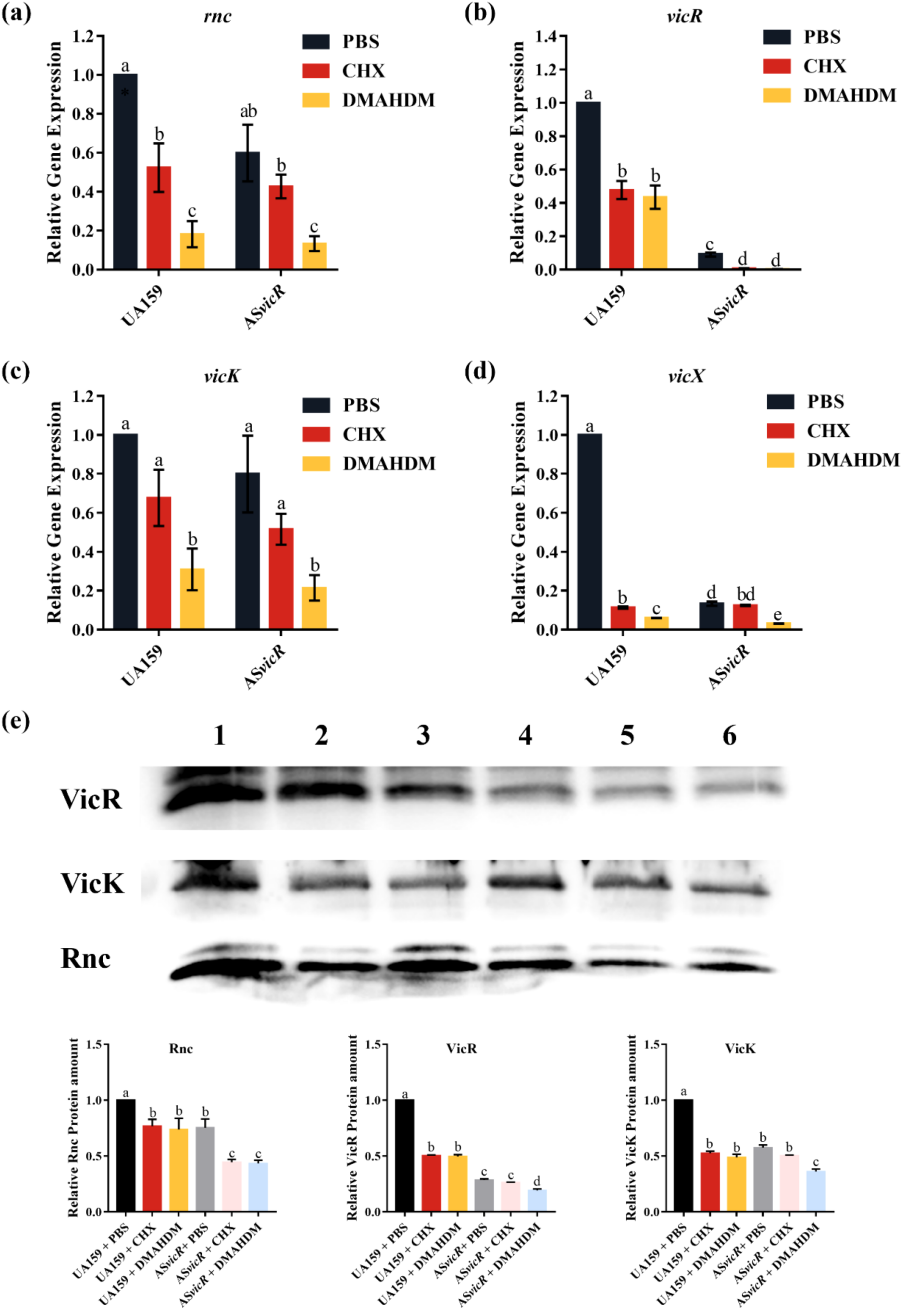
AS*vicR* combined with DMAHDM regulates the expression of genes and proteins of VicRK TCS. Gene expression of **(a)***rnc*, **(b)***vicR*, **(c)***vicK*, and **(d)***vicX* explored by RT-qPCR (n = 3); **(e)** protein expression levels of Rnc, VicR, and VicK (n = 3). Coomassie-stained gels confirmed equal protein loading among samples (see **Supplementary Figure 2**). Dissimilar letters represent significant differences (p < 0.05), 1: UA159 + PBS, 2: UA159 + CHX, 3: UA159 + DMAHDM, 4: AS*vicR* + PBS, 5: AS*vicR* + CHX, 6: AS*vicR* + DMAHDM.

Since the activity of Gtfs enzymes is closely associated with the VicRK TCS, we also investigated the expression of related genes. We found that in the AS*vicR* + DMAHDM group, not only was the *vicR* gene significantly downregulated, but *vicK*, *vicX*, and *rnc* were also markedly downregulated (**Figures 6a–d**). Consistent with genetic findings, western blot analysis revealed the weakest band intensity in the AS*vicR* + DMAHDM group compared to all other groups (**Figure 6e**). These results suggest that the combined application of AS*vicR* and DMAHDM may inhibit downstream EPS metabolism by modulating the VicRK TCS in *S. mutans* at both transcriptional and translational levels.

### 3.7 AS*vicR* combined with DMAHDM suppresses cariogenic pathogenicity *in vivo*

**Figure 7:**
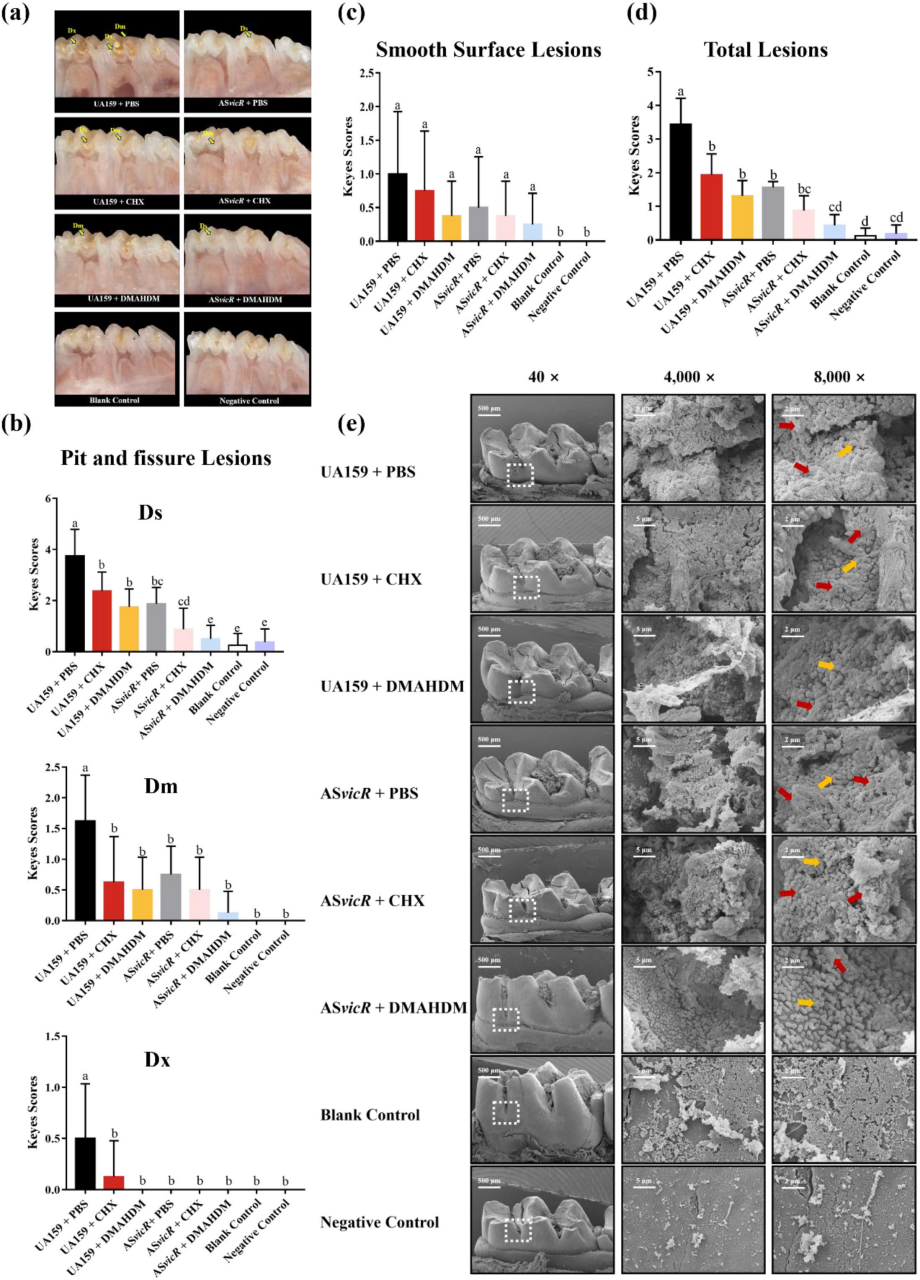
Combined treatment modulates the cariogenicity of *S. mutans* biofilms. **(a)** Representative stereomicroscopic images of rat mandibular molars; Keyes caries scores for **(b)** pit and fissure surfaces and **(c)** smooth surfaces, along with **(d)** the total caries score (n = 8); **(e)** SEM shows the architecture of plaque biofilms on rat mandibular molars. Dissimilar letters represent significant differences (p < 0.05).

**Figure 8:**
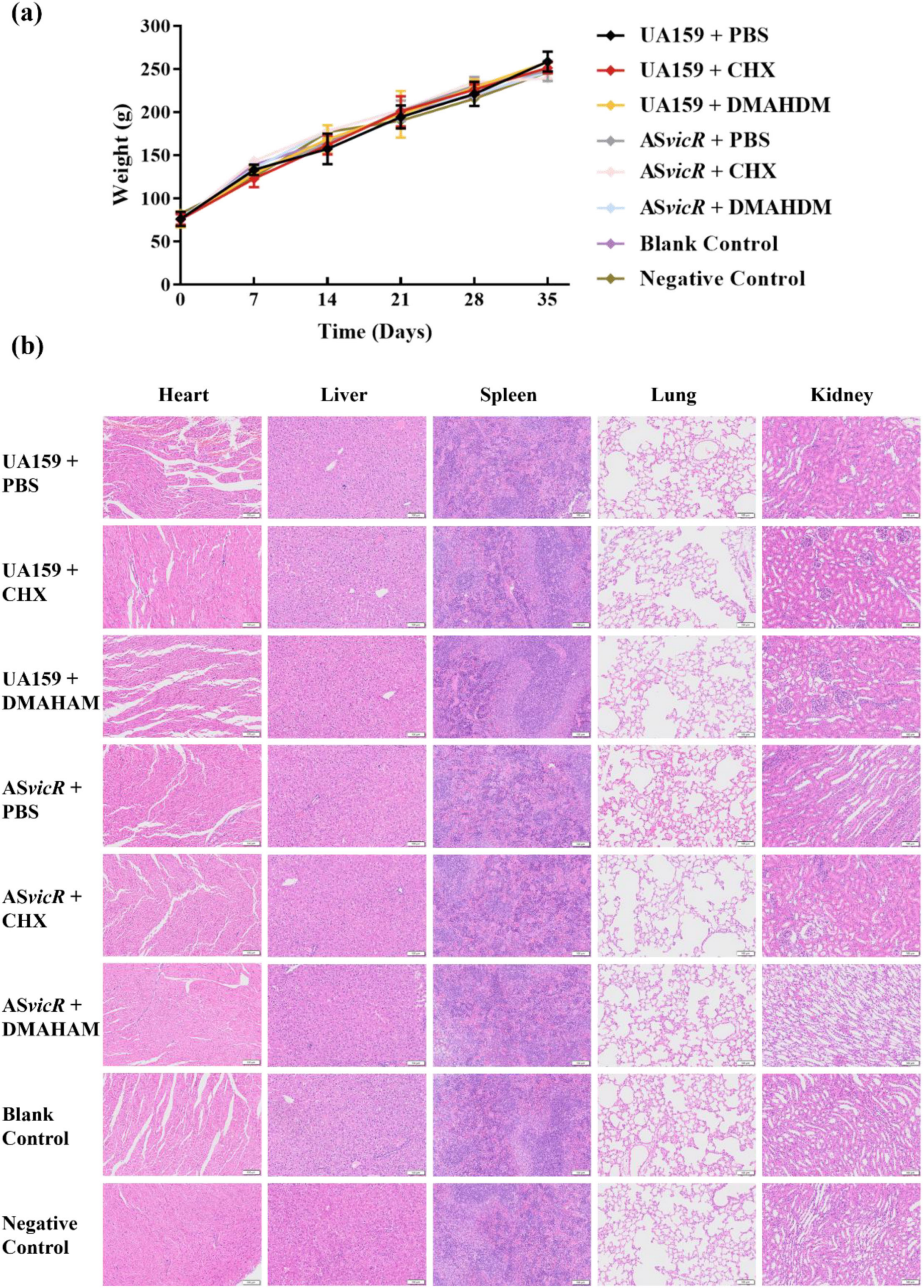
Toxicological evaluation of local drug administration. **(a)** Body weight steadily increased during different treatment periods (n = 8); **(b)** representative H&E staining images of major organs of rats in different groups.

Next, we established the rat caries model (see **Supplementary Figure 3**) to evaluate the suppression effect of AS*vicR* combined with DMAHDM on cariogenicity. The wild-type UA159 group exhibited the highest cariogenicity, with severe lesions across the Ds, Dm, and Dx regions (**Figure 7a**). In contrast, the AS*vicR* + DMAHDM group showed a marked reduction in caries severity, with lesion areas in the Ds, Dm, and Dx regions reduced to 13.3%, 7%, and 0% of the control group, respectively (**Figure 7b**). The smooth surface caries severity score showed no significant difference (**Figure 7c**). The total caries severity score decreased to 12.7% of the control (**Figure 7d**). SEM analysis of plaque biofilms revealed substantial structural alterations, with the AS*vicR* + DMAHDM group displaying markedly reduced EPS production, disrupted architecture, and a looser biofilm structure (**Figure 7e**). Toxicological assessment indicated no adverse effects. All rats exhibited steady weight gain during the one-month experiment (**Figure 8a**), and H&E staining results revealed no significant histopathological abnormalities in any treatment group (**Figure 8b**).

## 4 Discussion

Suppressing the cariogenic virulence factors represents an effective strategy for caries prevention. We demonstrated that the combined treatment of AS*vicR* and DMAHDM can significantly reduce CFU in the biofilm, lactate and EPS production and adversely affect biofilm biomass and architecture. In animal model, this approach markedly weakened both the number and severity of caries lesions. Consequently, this study proposes a “dual-targeting” model—achieving comprehensive inhibition of *S. mutans* through the action of disrupting bacterial signaling systems and membrane structure.

*S. mutans* produces extracellular matrix, which encapsulates itself and other microorganisms, to form dense biofilms (Su et al., 2025; Zhou et al., 2020) that limit the penetration of DMAHDM and attenuate its contact-based antibacterial efficacy (Jakubovics et al., 2021; Gao et al., 2016). The AS*vicR* overexpression strain showed reduced EPS and biofilm biomass, consistent with previous findings (Lei et al., 2020). The decrease in EPS likely alters biofilm permeability; the resulting porous and loosely structured biofilm facilitates enhanced penetration and action of DMAHDM. This structural alteration likely accounts for the pronounced reduction in biofilm CFU with the combined strategy.

DMAHDM has been reported to disrupt bacterial membrane potential, elevate osmotic pressure and induce cell lysis (Yu et al., 2024). Our results further demonstrated that DMAHDM treatment significantly elevated intracellular ROS levels in biofilm cells, with this effect amplified by AS*vicR* overexpression. This suggests involvement of the VicRK system in ROS stress responses. AS*vicR* overexpression likely impairs the normal post-transcriptional regulatory function of VicR, disrupting the bacterial capacity to manage extracellular stress and thereby increasing susceptibility to DMAHDM-induced oxidative damage (Qiu et al., 2025). This effect enhances the antibacterial efficacy of DMAHDM, aligning with reports by Qiu et al. that the VicRK system modulates oxidative stress tolerance, cell wall homeostasis, and antioxidant defense. These findings collectively indicate that the VicRK system plays a central role in biofilm formation and external antibacterial stressors. Consistent with this, live/dead staining revealed a highest proportion of dead bacteria in the DMAHDM + AS*vicR* group likely reflecting combined effects of DMAHDM-induced ROS accumulation and AS*vicR*-mediated attenuation of antioxidant responses, leading to excessive ROS and cell death.

Biofilm in the AS*vicR* + DMAHDM group displayed minimal structural formation, with bacteria appearing as scattered individual cells and markedly decreased EPS levels. These effects are likely related to the regulation of two key enzymes in EPS metabolism: glucosyltransferases (Gtfs), responsible for EPS synthesis, and dextranase (DexA) (Cugini et al., 2019), which degrades EPS. RT-qPCR analysis of related gene expression, zymographic assessment of *Gtfs* activity, and Western blot detection of DexA protein levels supported this hypothesis. Specifically, *GtfB and GtfC* primarily synthesizes WIG, promoting bacterial aggregation and forming the scaffold essential for stability and integrity; *GtfD* generates WSG, providing adhesion sites and an energy source for bacteria. The combined AS*vicR* + DMAHDM treatment significantly reduced the production of both WIG and WSG. Concurrently, downregulation of *gbpB* and *gbpC* diminished the glucan-dependent co-aggregation ability of *S. mutans* (Zhu et al., 2009), weakening bacterial adhesion and biofilm cohesion, leading to a porous and loosely structured biofilm (Kashi et al., 2025). Furthermore, DexA (Yang et al., 2019) was upregulated in the AS*vicR* + DMAHDM group, not only facilitating glucan degradation and biofilm matrix remodeling but also attenuating the bacterial stress response. Consequently, the biofilm-forming capacity was weakened, and its adaptability to environmental changes decreased, reducing its cariogenic potential—consistent with SEM and CLSM observations. In summary, the combined intervention of AS*vicR* and DMAHDM suppresses EPS synthesis while accelerating its degradation and clearance. This “dual regulatory effect”—simultaneously inhibiting synthesis and promoting degradation—continuously weakens the glycosidic network within the biofilm, thereby reducing bacterial adhesion and leading to structural disintegration. This mechanism elucidates, from a bidirectional regulatory perspective, the observed reduction in both biofilm biomass and overall cariogenicity.

Previous studies have established that EPS metabolism is closely associated with the VicRK TCS. Consistent with our research results, AS*vicR* inhibits the translation of the VicR protein through complementary binding to *vicR* mRNA, subsequently leading to the downregulation of downstream genes (Lei et al., 2020). VicK, a membrane-bound sensor kinase, perceives signals such as cell wall damage or oxidative stress and phosphorylates VicR. The expression of the *vicK* gene in the AS*vicR* + PBS group did not show significant downregulation, but a decrease was observed after DMAHDM treatment. This reduction may be related to the disruption of membrane potential and consequent breakdown of cell wall homeostasis induced by DMAHDM (Chen et al., 2024). The *rnc* gene, located upstream of the *vicRK* operon, is involved in the virulence regulation of *S. mutans* biofilms. We found that compared to the AS*vicR* + PBS group, the AS*vicR* + DMAHDM group further significantly downregulated the *vicR* and *rnc* genes and reduced the protein expression level. These results suggest that DMAHDM, potentially through membrane stress interference, disrupts the normal sensing and transduction functions of the VicRK two-component system, thereby amplifying the inhibitory effect of AS*vicR* on *vicR*. This leads to a dual inhibition—both chemical and genetic—resulting in further downregulation of the key regulatory protein VicR. This dual inhibitory action causes further disruption of bacterial cell wall homeostasis, a greater decrease in the expression of adhesion-related genes (e.g., *gtfB/C/D*, *gbpB*), ultimately weakening biofilm-forming capacity and affecting bacterial survival. Given that *vicR* is essential for *S. mutans* viability, the enhanced suppression observed in the AS*vicR* + DMAHDM group suggests a damaging effect. This finding not only reveals a potential target of DMAHDM but also supports its superior antibiofilm properties compared to conventional antimicrobial agents like CHX at a molecular level.

Subsequently, we employed a rat caries model to simulate the interactions between the host, microorganisms, and diet *in vivo*. Notably, compared to the wild-type group, the AS*vicR* + DMAHDM group significantly reduced the occurrence of extensive (Dx) and moderate (Dm) dentinal caries, indicating a strong inhibitory effect on demineralization and suggesting its potential as a caries management strategy. SEM further confirmed that the dense structure of the biofilm was disintegrated and the EPS content was decreased in the AS*vicR* + DMAHDM group. Previous studies reported that deletion of genes related to *gtfB*/*C*/*D* or *vicR/K* impairs bacterial signal transduction ability and reduces the *in vivo* cariogenicity of biofilms, which supports the observed reduction in the severity of caries in the AS*vicR* + DMAHDM group (Sun et al., 2023). Therefore, gene therapy approaches based on AS*vicR* overexpression, combined with DMAHDM, show promise as a valuable supplement to conventional caries prevention methods, particularly for caries-susceptible individuals or those with poor oral hygiene.

Although this research provides valuable biological insights, it also has certain limitations. The experimental design relied on a single-species biofilm model, which may not fully recapitulate the complex interactions occurring in a multi-species oral environment. Furthermore, although we observed a positive correlation between elevated ROS levels and biofilm inhibition, the causal relationship requires further elucidation. Subsequent studies employing ROS scavengers or constructing specific stress-response gene mutants would help establish the underlying mechanistic connections. The strategy of combining AS*vicR* overexpression with DMAHDM exerts its effects through interference with the VicRK TCS in *S. mutans*, ultimately inhibiting the various cariogenic virulence factors of biofilm, thereby reducing its cariogenic potential. This sequential cascade of “signal modulation–metabolic remodeling–structural disruption” highlights a conceptual dual-targeting framework for caries control. In this context, DMAHDM could be incorporated into dental materials or oral care products to provide sustained, contact-active antibacterial effects, whereas AS*vicR* -based approaches are envisioned as gene-targeting tools that would require appropriate nucleic acid delivery systems, such as nanoparticle platforms or tetrahedral framework nucleic acids. This work provides new scientific evidence for developing antibacterial materials based on bacterial signaling pathway regulation, offering novel insights for mechanism-based caries prevention strategies.

## Data Availability

The original contributions presented in the study are included in the article/**Supplementary Material**. Further inquiries can be directed to the corresponding authors.

## References

[B1] Balhaddad A. A. Garcia I. M. Mokeem L. (2021). Bifunctional composites for biofilms modulation on cervical restorations. J. Dental Res. 100, 1063–1071. doi: 10.1177/00220345211018189, PMID: 34167373

[B2] Bustin S. A. (2024). Improving the quality of quantitative polymerase chain reaction experiments: 15 years of MIQE. Mol. Aspects Med. 96, 101249. doi: 10.1016/j.mam.2024.101249, PMID: 38290180

[B3] Chen H. Tang Y. Weir M. D. (2020). Effects of S. mutans gene-modification and antibacterial monomer dimethylaminohexadecyl methacrylate on biofilm growth and acid production. Dental Materials 36, 296–309. doi: 10.1016/j.dental.2019.12.001, PMID: 31839202

[B4] Chen H. Xu M. Zhang B. (2024). Novel strategy of S. mutans gcrR gene over-expression plus antibacterial dimethylaminohexadecyl methacrylate suppresses biofilm acids and reduces dental caries in rats. Dental Materials 40, e41–e51. doi: 10.1016/j.dental.2024.06.018, PMID: 38942710

[B5] Chen R. Du M. Liu C. (2022). Strategies for dispersion of cariogenic biofilms: applications and mechanisms. Front. Microbiol. 13, 981203. doi: 10.3389/fmicb.2022.981203, PMID: 36134140 PMC9484479

[B6] Chen Z. Zhao X. Zheng H. Wang Y. Zhang L. (2025). Advances and challenges in drug design against dental caries: Application of in silico approaches. J. Pharm. Anal. 15, 101161. doi: 10.1016/j.jpha.2024.101161, PMID: 40678483 PMC12268077

[B7] Collaborators G. O. D. (2025). Trends in the global, regional, and national burden of oral conditions from 1990 to 2021: a systematic analysis for the Global Burden of Disease Study 2021. Lancet 405, 897–910. doi: 10.1016/S0140-6736(24)02811-3, PMID: 40024264

[B8] Cugini C. Shanmugam M. Landge N. Ramasubbu N. (2019). The role of exopolysaccharides in oral biofilms. J. Dental Res. 98, 739–745. doi: 10.1177/0022034519845001, PMID: 31009580 PMC6589894

[B9] Deng Y. Yang Y. Zhang B. (2021). The vicK gene of Streptococcus mutans mediates its cariogenicity via exopolysaccharides metabolism. Int. J. Oral. Sci. 13, 45. doi: 10.1038/s41368-021-00149-x, PMID: 34916484 PMC8677823

[B10] Du H. Wang Z. Long S. Li Y. Yang D. (2025). The advancement of nanosystems for drug delivery in the prevention and treatment of dental caries. Front. Cell. Infection Microbiol. 15, 1546816. doi: 10.3389/fcimb.2025.1546816, PMID: 40007606 PMC11850577

[B11] Emmanuelli B. Knorst J. K. Menegazzo G. R. Mendes F. M. Ardenghi T. M. (2023). Dental caries prediction and the indication of pit and fissure sealant in children first permanent molars: A prospective study. J. Dentistry 135, 104557. doi: 10.1016/j.jdent.2023.104557, PMID: 37230242

[B12] Fanfoni L. Marsich E. Turco G. Breschi L. Cadenaro M. (2021). Development of di-methacrylate quaternary ammonium monomers with antibacterial activity. Acta Biomaterialia 129, 138–147. doi: 10.1016/j.actbio.2021.05.012, PMID: 34023457

[B13] Gao X. Jiang S. Koh D. Hsu C. Y. (2016). Salivary biomarkers for dental caries. Periodontology 2000 70, 128–141. doi: 10.1111/prd.12100, PMID: 26662487

[B14] Gonzalez N. Sevillano D. Alou L. (2013). Influence of the MBC/MIC ratio on the antibacterial activity of vancomycin versus linezolid against methicillin-resistant Staphylococcus aureus isolates in a pharmacodynamic model simulating serum and soft tissue interstitial fluid concentrations reported in diabetic patients. J. Antimicrobial Chemotherapy 68, 2291–2295. doi: 10.1093/jac/dkt185, PMID: 23674766

[B15] Hemadi A. S. Huang R. Zhou Y. Zou J. (2017). Salivary proteins and microbiota as biomarkers for early childhood caries risk assessment. Int. J. Oral. Sci. 9, e1. doi: 10.1038/ijos.2017.35, PMID: 29125139 PMC5775330

[B16] Jakubovics N. S. Goodman S. D. Mashburn-Warren L. Stafford G. P. Cieplik F. (2021). The dental plaque biofilm matrix. Periodontology 2000 86, 32–56. doi: 10.1111/prd.12361, PMID: 33690911 PMC9413593

[B17] Jiang Q. Xu M. Chen H. (2025). V-ATPase contributes to the cariogenicity of Candida albicans- Streptococcus mutans biofilm. NPJ Biofilms Microbiomes 11, 41. doi: 10.1038/s41522-025-00660-7, PMID: 40057552 PMC11890576

[B18] Kashi M. Varseh M. Hariri Y. Chegini Z. Shariati A. (2025). Natural compounds: new therapeutic approach for inhibition of Streptococcus mutans and dental caries. Front. Pharmacol. 16, 1548117. doi: 10.3389/fphar.2025.1548117, PMID: 40235544 PMC11996897

[B19] Kawada-Matsuo M. Komatsuzawa H. (2017). Role of Streptococcus mutans two-component systems in antimicrobial peptide resistance in the oral cavity. Japanese Dental Sci. Rev. 53, 86–94. doi: 10.1016/j.jdsr.2016.12.002, PMID: 28725299 PMC5501732

[B20] Lei L. Stipp R. N. Chen T. Wu S. Z. Hu T. Duncan M. J. (2018). Activity of streptococcus mutans vicR is modulated by antisense RNA. J. Dental Res. 97, 1477–1484. doi: 10.1177/0022034518781765, PMID: 29969955 PMC6262263

[B21] Lei L. Zhang B. Mao M. (2020). Carbohydrate metabolism regulated by antisense vicR RNA in cariogenicity. J. Dental Res. 99, 204–213. doi: 10.1177/0022034519890570, PMID: 31821772

[B22] Lei L. Zhang Y. Xu Y. (2023). Spermine-starch nanoparticles with antisense vicR suppress Streptococcus mutans cariogenicity. J. Materials Chem. B 11, 5752–5766. doi: 10.1039/D2TB02628G, PMID: 37219356

[B23] Lin G. S. S. Cher C. Y. Cheah K. K. Noorani T. Y. Ismail N. H. Ghani N. R. N. A. (2022). Novel dental composite resin derived from rice husk natural biowaste: A systematic review and recommendation for future advancement. J. Esthetic Restorative Dentistry 34, 503–511. doi: 10.1111/jerd.12831, PMID: 34716755

[B24] Liu C. Tan D. Chen X. Liao J. Wu L. (2022). Research on graphene and its derivatives in oral disease treatment. Int. J. Mol. Sci. 23, 4737. doi: 10.3390/ijms23094737, PMID: 35563128 PMC9104291

[B25] Liu H. Yue Y. Xu Z. Guo L. Wu C. Zhang D. . (2023). mTORC1 signaling pathway regulates tooth repair. Int. J. Oral. Sci. 15, 14. doi: 10.1038/s41368-023-00218-3, PMID: 36927863 PMC10020452

[B26] Liu S. Tao Y. Yu L. (2016). Analysis of Small RNAs in Streptococcus mutans under Acid Stress-A New Insight for Caries Research. Int. J. Mol. Sci. 17, 1529. doi: 10.3390/ijms17091529, PMID: 27649155 PMC5037804

[B27] Qiu B. Deng Y. Yi Z. Yang Y. Lei L. Hu T. (2025). Harnessing the regulatory effects of streptococcus mutans two-component signal transduction systems for therapeutic interventions against dental caries. Mol. Oral. Microbiol. 40, 243–257. doi: 10.1111/omi.70006, PMID: 40863791

[B28] Quintero-Yanes A. Petit K. Rodriguez-Villalobos H. (2025). Multiplexed bacteriocin synthesis to combat and prevent antimicrobial resistance. Commun. Biol. 8, 1246. doi: 10.1038/s42003-025-08639-y, PMID: 40830650 PMC12365309

[B29] Selwitz R. H. Ismail A. I. Pitts N. B. (2007). Dental caries. Lancet 369, 51–59. doi: 10.1016/S0140-6736(07)60031-2, PMID: 17208642

[B30] Su C. Li C. Sun K. Li W. Liu R. (2020). Quantitative analysis of bioactive components in walnut leaves by UHPLC-Q-Orbitrap HRMS combined with QAMS. Food Chem. 331, 127180. doi: 10.1016/j.foodchem.2020.127180, PMID: 32544651

[B31] Su C. Zhu M. Guo Y. (2025). DMAHDM@MPC nanoparticles in orthodontic adhesive inhibit cariogenic bacteria and sugar metabolism to prevent enamel demineralization. Materials Today Bio 33, 101969. doi: 10.1016/j.mtbio.2025.101969, PMID: 40547485 PMC12182376

[B32] Sugai K. Kawada-Matsuo M. Nguyen-Tra Le M. (2023). Isolation of Streptococcus mutans temperate bacteriophage with broad killing activity to S. mutans clinical isolates. iScience 26, 108465. doi: 10.1016/j.isci.2023.108465, PMID: 38089578 PMC10713843

[B33] Sun Y. Chen H. Xu M. (2023). Exopolysaccharides metabolism and cariogenesis of Streptococcus mutans biofilm regulated by antisense vicK RNA. J. Oral. Microbiol. 15, 2204250. doi: 10.1080/20002297.2023.2204250, PMID: 37138664 PMC10150615

[B34] Tian Y. Zhang Y. Zhang M. Chen X. Lei L. Hu T. (2022). Antisense vicR-Loaded Dendritic Mesoporous Silica Nanoparticles Regulate the Biofilm Organization and Cariogenicity of Streptococcus mutans. Int. J. Nanomedicine 17, 1255–1272. doi: 10.2147/IJN.S334785, PMID: 35340824 PMC8956320

[B35] Wang S. Wang H. Ren B. (2017). Do quaternary ammonium monomers induce drug resistance in cariogenic, endodontic and periodontal bacterial species? Dental Materials 33, 1127–1138. doi: 10.1016/j.dental.2017.07.001, PMID: 28755761

[B36] Wang X. Zhao Y. Qi X. (2022). Quantitative analysis of metabolites in the aflatoxin biosynthesis pathway for early warning of aflatoxin contamination by UHPLC-HRMS combined with QAMS. J. Hazardous Materials 431, 128531. doi: 10.1016/j.jhazmat.2022.128531, PMID: 35220124

[B37] Wiegand I. Hilpert K. Hancock R. E. (2008). Agar and broth dilution methods to determine the minimal inhibitory concentration (MIC) of antimicrobial substances. Nat. Protoc. 3, 163–175. doi: 10.1038/nprot.2007.521, PMID: 18274517

[B38] Yang Y. Mao M. Lei L. (2019). Regulation of water-soluble glucan synthesis by the Streptococcus mutans dexA gene effects biofilm aggregation and cariogenic pathogenicity. Mol. Oral. Microbiol. 34, 51–63. doi: 10.1111/omi.12253, PMID: 30659765

[B39] Yu S. Xu M. Wang Z. Deng Y. Xu H. Weir M. D. . (2024). S. mutans Antisense vicK RNA Over-Expression Plus Antibacterial Dimethylaminohexadecyl Methacrylate Suppresses Oral Biofilms and Protects Enamel Hardness in Extracted Human Teeth. Pathogens 13, 707. doi: 10.3390/pathogens13080707, PMID: 39204307 PMC11356802

[B40] Zhang H. Xia M. Zhang B. (2022). Sucrose selectively regulates Streptococcus mutans polysaccharide by GcrR. Environ. Microbiol. 24, 1395–1410. doi: 10.1111/1462-2920.15887, PMID: 35064734

[B41] Zhou W. Peng X. Zhou X. (2020). *In vitro* evaluation of composite containing DMAHDM and calcium phosphate nanoparticles on recurrent caries inhibition at bovine enamel-restoration margins. Dental Materials 36, 1343–1355. doi: 10.1016/j.dental.2020.07.007, PMID: 32800353

[B42] Zhu M. Ajdić D. Liu Y. Lynch D. Merritt J. Banas J. A. (2009). Role of the Streptococcus mutans irvA gene in GbpC-independent, dextran-dependent aggregation and biofilm formation. Appl. Environ. Microbiol. 75, 7037–7043. doi: 10.1128/AEM.01015-09, PMID: 19783751 PMC2786544

