# Supplementary material for: Overexpression of ASvicR combined with the antibacterial monomer DMAHDM interferes with the VicRK two-component system to attenuate the cariogenicity of Streptococcus mutans
Source: Front Cell Infect Microbiol. 2026 Mar 5;16:1793140. doi: 10.3389/fcimb.2026.1793140 (PMC12999838; doi:10.3389/fcimb.2026.1793140)
Supplement: Supplementary file 1 [file Table1.docx]

Supplementary Material

**Table 1. Bacterial strains and plasmids used in this study.**

| Strains or Plasmids | Description | Source or reference |
| --- | --- | --- |
| Parent *S. mutans* | UA159 | ^a^ATCC 700610 |
| AS*vicR* *S. mutans* | AS*vicR* over expression strain | [1] |

^a^ATCC: American Type Culture Collection.

**Table 2. Sequences of primers used for RT-qPCR.**

| Primer | Nucleotide sequence | Source or Reference |
| --- | --- | --- |
| *gyrA-F* | 5' ATTGTTGCTCGGGCTCTTCCAG 3' | [1] |
| *gyrA-R* | 5' ATGCGGCTTGTCAGGAGTAACC 3' | [1] |
| *vicR-F* | 5' CGCAGTGGCTGAGGAAAATG 3' | [1] |
| *vicR-R* | 5' ACCTGTGTGTGTCGCTAAGTGATG 3' | [1] |
| *gtfB-F* | 5' ACACTTTCGGGTGGCTTG 3' | [1] |
| *gtfB-R* | 5' GCTTAGATGTCACTTCGGTTG 3' | [1] |
| *gtfC-F* | 5' CCAAAATGGTATTATGGCTGTCG 3' | [1] |
| *gtfC-R* | 5' TGAGTCTCTATCAAAGTAACGCAG 3' | [1] |
| *gtfD-F* | 5' AATGAAATTCGCAGCGGACTTGAG 3' | [1] |
| *gtfD-R* | 5' TTAGCCTGACGCATGTCTTCATTGTA 3' | [1] |
| *ftf-F* | 5' ATTGGCGAACGGCGACTTACTC 3' | [1] |
| *ftf-R* | 5' CCTGCGACTTCATTACGATTGGTC 3' |  |
| *gbpb-F* | 5' ATGGCGGTTATGGACACGTT 3' | [1] |
| *gbpb-R* | 5' TTTGGCCACCTTGAACACCT 3' | [1] |
| *gbpc-F* | 5' AGAGAAAGCACTTTGGTTTCAATTGGAACT 3' | [1] |
| *gbpc-R* | 5' GGGCGGAACACTTTTTTGTCCCAACCTCTT 3' | [1] |
| *dexA-F* | 5' AGGGCTGACTGCTTCTGGAGT 3' | [1] |
| *dexA-R* | 5' AGTGCCAAGACTGACGCTTTG 3' | [1] |
| *dexB-F* | 5' AGAACACCTATGCAATGGGATGCTT 3' | [1] |
| *dexB-R* | 5' GTTGCTGAATGAGTTGTTGATAGGT 3' | [1] |
| *vicK-F* | 5' CACTTTACGCATTCGTTTTGCC 3' | [1] |
| *vicK-R* | 5' CGTTCTTCTTTTTCCTGTTCGGTC 3' | [1] |
| *vicX-F* | 5' TGCTCAACCACAGTTTTACCG 3' | [1] |
| *vicX-R* | 5' GGACTCAATCAGATAACCATCAGC 3' | [1] |
| *rnc-F* | 5' CAGCCTCTTGCTCTGCTAATTTT 3' | [1] |
| *rnc-R* | 5' AAGTTGACGGGGATGTTTTGAT 3' | [1] |

**Figure 1. Coomassie-stained gels confirmed equal protein loading among samples.**


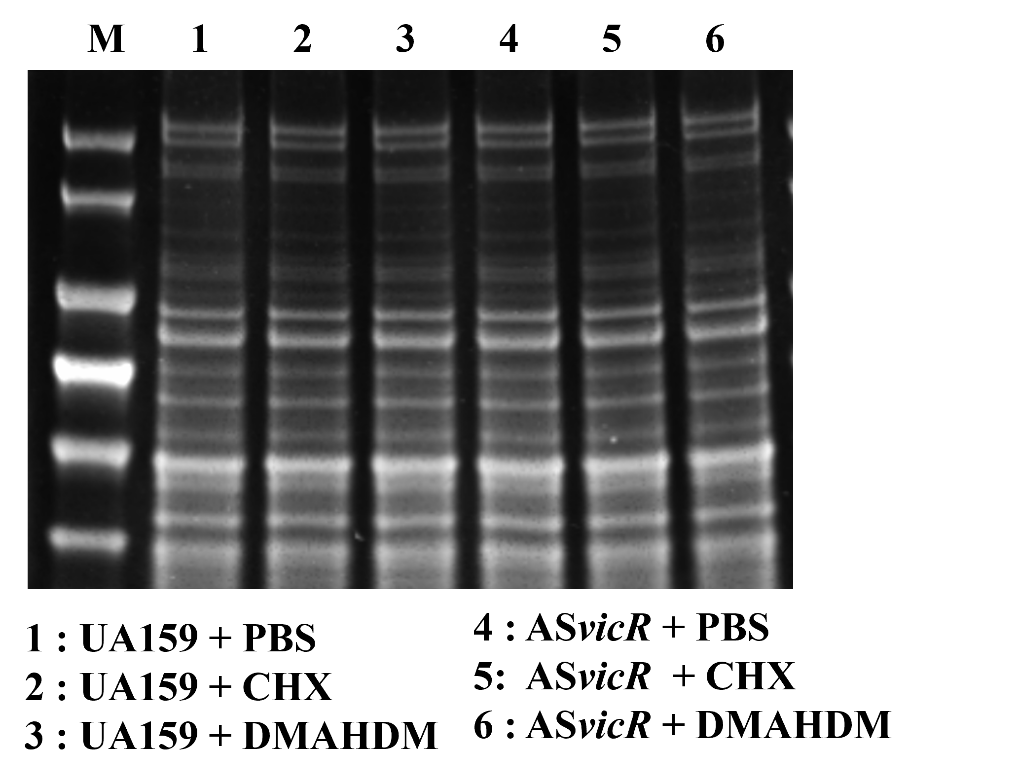


**Figure 2. Coomassie-stained gels confirmed equal protein loading among samples.**

**
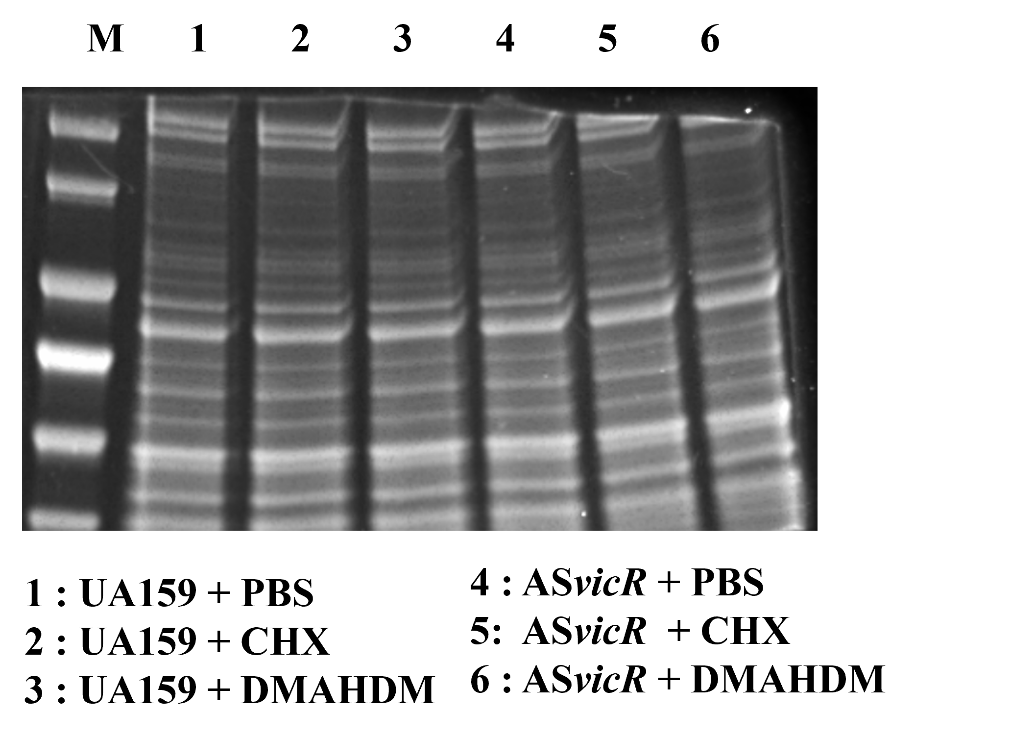
**


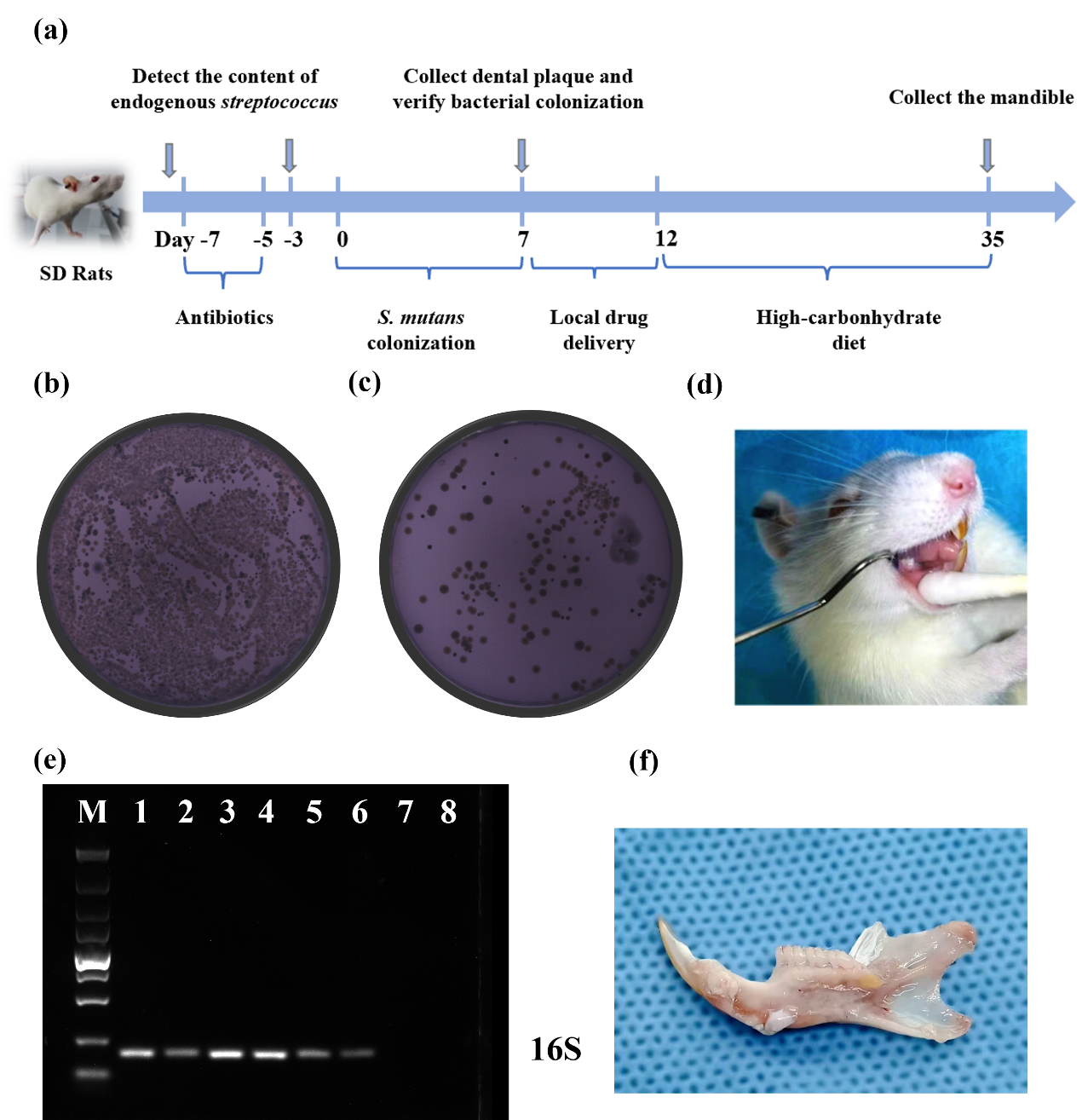


**Figure 3. Rat caries model establishment and validation.​ (a) Schematic diagram of the experimental workflow; oral streptococcal levels (b) before and (c) after antibiotic treatment, determined by colony counting on MSA plates; (d) inoculation of cariogenic bacteria onto the mandibular molar surfaces; (e) PCR verification of successful *S. mutans* colonization on rat mandibular molars; (f) intact mandibular molars harvested for analysis.**

**Reference**

[1]Lei L, Zhang B, Mao M. Carbohydrate Metabolism Regulated by Antisense vicR RNA in Cariogenicity. Journal of Dental Research. 2020;99(2):204-13.
